# Walking cadence as a measure of activity intensity and impact on functional capacity for prefrail and frail older adults

**DOI:** 10.1371/journal.pone.0323759

**Published:** 2025-07-16

**Authors:** Daniel S. Rubin, Anthony Hung, Emi Yamamoto, Donald Hedeker, David E. Conroy, Megan Huisingh-Scheetz, Jennifer S. Brach, Nancy W. Glynn, Margaret K. Danilovich

**Affiliations:** 1 Department of Anesthesia and Critical Care, University of Chicago, Chicago, Illinois, United States of America; 2 Pritzker School of Medicine, University of Chicago, Chicago, Illinois, United States of America; 3 Department of Public Health Sciences, University of Chicago, Chicago, Illinois, United States of America; 4 School of Kinesiology, University of Michigan. Ann Arbor, Michigan, United States of America; 5 Department of Medicine, Section of Geriatrics, University of Chicago, Chicago, Illinois, United States of America; 6 Department of Physical Therapy, University of Pittsburgh, Pittsburgh, Pennsylvania, United States of America; 7 School of Public Health, Department of Epidemiology, University of Pittsburgh, Pittsburgh, Pennsylvania, United States of America; 8 Leonard Schanfield Research Institute, CJE Senior Life, Chicago, Illinois, United States of America; Hamasaki Clinic, JAPAN

## Abstract

Walking cadence has been suggested as a measure of activity intensity; however, it remains uncertain if prefrail and frail older adults can increase their walking cadence and if doing so leads to improvements in functional capacity. We aimed to determine if cadence can be increased and if this leads to improvement in functional capacity in prefrail and frail older adults. We performed a secondary data analysis of a walking intervention in prefrail and frail older adults living in retirement communities. Patients were randomized to Casual Speed Walking (CSW) and High-Intensity Walking (HIW) groups. Our primary outcome was improvement in 6-minute walk test distance above the minimally clinical important difference. We performed linear and logistic mixed-effects regressions to analyze our aims. 102 participants were included in the final analysis with 56 in the CSW group and 46 in the HIW group. Participants in the HIW group increased their walking cadence as compared to the CSW group during the intervention (HIW 100[88, 111] steps/min vs. CSW 77[65, 86] steps/min; P < 0.001). Participants that increased their walking cadence demonstrated an increased odds of improvement in their 6-minute walk test minimum clinically important difference (OR: 0.11, 95% CI: 0.033, 0.18; p = 0.005). Older adults can increase their walking cadence and walking cadence can serve as a surrogate measure of activity intensity during walking interventions. An increase of 14 steps/minute from their comfortable walking cadence increased the odds of improvement in 6-minute walk test minimum clinically important difference.

## Introduction

Frailty is an age-related syndrome of increased vulnerability to stressors that is associated with poor health outcomes including falls, incident disability, hospitalization, and death [1,2]. The frailty phenotype consists of five different domains: weight loss, slowness, weakness, exhaustion, and low physical activity [1]. Importantly, four of the five frailty domains directly relate to physical activity which makes exercise a natural therapeutic intervention to improve functional capacity among older adults with frailty [3].

Walking is an important exercise intervention to reduce frailty in older adults as it addresses the frailty criteria of slowness, low physical activity and exhaustion [4,5]. Walking directly benefits the circulatory, cardiopulmonary and immune systems while reducing the risk and severity of various health conditions, including cardiovascular disease, cerebrovascular disease, cognitive impairment, dementia and sleep disturbances [6]. Further, aerobic capacity is decreased in frailty beyond normal age-related changes, making activity of daily living completion difficult as these tasks require the person to operate at a near-maximum capacity [7]. A VO_2_max of 18 ml/kg/min or greater is required to complete most activities of daily living tasks, yet research estimates frail older adults have VO_2_ values under 16 ml/kg/min [8,9]. Walking for exercise can improve aerobic capacity and potentially reduce the frailty syndrome [10,11]. From a clinical and public health perspective, walking interventions are advantageous as they are easily scalable, low cost, can be performed in different environments with no additional resources or equipment, and can be completed by older adults possessing different levels of skill and functional abilities [12,13].

The concept of an exercise dose includes three different domains that include the frequency, duration, and intensity [14]. A strong body of evidence supports a dose-response effect between a dose of exercise and improvements in functional capacity [15–18]. Objective measurements of an exercise dose would facilitate dose titration to advance health outcomes. Exercise frequency and duration are easy to describe and measure as they correspond to the number of days a week and amount of time a single session of exercise is performed, respectively. Exercise intensity is more challenging to describe and measure, as commonly employed methods, such as heart rate, rating of perceived exertion (RPE), and the talk test are prone to inaccuracy and there are notable limitations for all these methods [19,20]. Older adults are commonly on medications (i.e., beta blockers) that limit increases in heart rate in response to exercise and limit its utility as an intensity measure in this population. RPE and the talk test are subjective assessments made by the individual and older adults have difficulty correctly identifying an activity intensity associated with a given RPE [21,22].

Recently, walking cadence, defined as the number of steps walked for a unit of time, has increased in popularity as a standardized and objective measure of activity intensity during walking [23,24]. While it remains unclear whether cadence changes with age, older adults with a higher cadence have lower odds of experiencing decreased walking speed [25,26]. Walking cadence can be objectively measured in real time by accelerometers, including those integrated into smartphones, making it an appealing measure to guide intensity during walking interventions [27]. However, while walking cadence has been associated with activity intensity and prescribed to increase moderate intensity walking, it has not been directly measured throughout a walking intervention to assess its association with improvements in functional capacity in prefrail and frail older adults [28–30]. Further, it remains unknown whether prefrail and frail older adults can increase their walking cadence in response to an exercise intervention.

To assess whether older adults can increase their walking cadence and whether cadence can serve as a surrogate for activity intensity in walking interventions, we conducted a secondary analysis of a clinical trial comparing high- versus low-intensity walking in prefrail and frail older adults. Here we report the accelerometer-recorded walking cadence among prefrail and frail older adults from walking sessions over the course of the 4-month trial. Our first objective was to determine if there were significant differences in median walking cadence by treatment group. We hypothesized that participants assigned to the high-intensity walking group would have a faster cadence over the study period as compared to the lower intensity walking group. Our second objective was to determine the impact of increases in participant-level walking cadence on improvements in functional capacity irrespective of group assignment. We hypothesized that participants who increased their cadence throughout the 4-month intervention would have an increased odds of improvement in functional capacity.

## Methods

### Study design

We performed a retrospective secondary data analysis investigating the effects of high- versus low-intensity walking on functional capacity, frailty, mobility, physical functioning, balance, and daily physical activity among prefrail and frail older adults (NCT03654807) [31]. Briefly, participants were randomized from November 2017 to April 2022 within 14 independent living retirement communities to either a: 1) high intensity walking regimen (HIW) or 2) casual speed walking regimen (CSW). The Northwestern University institutional review board approved the study protocol. All participants provided written informed consent, and all data were deidentified. The trial protocol can be found in S1 Table 1.

### Study sample and data collection

Older adults residing in independent living retirement communities in the Chicagoland area (urban and suburban) were recruited to participate. The retirement communities (n = 14) included a diverse racial and economic cohort of older adults. Recruitment was performed through presentations and flyers to residents. Older adults were eligible for the study if they were permanent residents in the retirement community, ≥ 60 years of age, were prefrail or frail according to the Survey of Health, Ageing, and Retirement in Europe Frailty Instrument (SHARE-FI) and were capable of ambulating at least 10 feet with moderate assistance (<50% physical assistance) or less. Exclusion criteria included uncontrolled cardiovascular, metabolic, renal, or respiratory disease that prevented safe exercise participation or a resting blood pressure > 180/110 mmHg.

### Assessments

Participants were assessed at the time of enrollment and following the 4-month intervention in their retirement community. Study procedures included demographic and medical history. The mini-mental state examination (MMSE) was used to identify the presence of cognitive dysfunction [32]. Frailty was measured using the SHARE-FI to determine the categorical level of frailty and the overall continuous frailty score. Objective measures of physical function included the Timed up and go (TUG), 4-meter Gait speed (usual and fast pace), Short Physical Performance Battery (SPPB), Berg Balance Scale (BBS) and 6-minute walk test (6MWT) [33–35]. Assessments were conducted by a trained physical therapist, masked to group assignment, following standardized testing procedures for all measures.

### Intervention

The intervention consisted of 48 total sessions; with 3 sessions performed per week for 4-months (S1 Table). Sessions were divided into three distinct phases: Phase 1 - acclimation (sessions 1–3), Phase 2 - ramp-up (sessions 4–12) and Phase 3 - intervention (sessions 13–48). Each walking session lasted approximately 45 minutes of which 15 minutes were allocated to warm-up and cool-down and 30 minutes to the intervention. Participants were able to take rest breaks as necessary and rest breaks were not counted towards the 30 minutes of total walking per session. For HIW and CSW, Phase 1 consisted of 3 sessions with 45 minutes of casual self-selected pace walking. For Phase 2, sessions consisted of 40 minutes of walking with 5 minutes of stair tapping or stepping at the beginning and end of the session (2.5 minutes at the beginning and 2.5 minutes at the end). Stair tapping and stepping were incorporated to simulate walking tasks encountered in the home and community. During Phase 2 HIW participants were encouraged to increase their intensity to reach 70% of heart rate maximum (HRmax) by session 12. Research assistants measured heart rate using a Polar A370 (Polar Electro, Kempele Finland) continuous heart rate monitor on the wrist to ensure each participant was walking within their individually calculated heart rate intensity. The CSW participants walked, and stair tapped or stepped at their own comfortable pace. For Phase 3, both groups performed a 10-minute warmup followed by 5-minute increments of walking, stair tapping or stepping, walking with weights, and walking in variable directions. The HIW participants were instructed to walk or step “as fast as they safely could,” throughout the sessions while the CSW were instructed to walk in a “relaxed and comfortable pace,” to achieve the targeted intensity. All sessions were performed in the participants retirement community and led by a trained research assistant who used a standardized script of motivational prompts to ensure standardization between groups.

### Cadence

Participants wore an activPAL accelerometer (PAL Technologies Ltd, Glasgow Scotland) on the anterior thigh throughout each session. The session data was downloaded and analyzed using PAL software (version 8.11.6.70) with the different walking tasks manually identified by research assistants. We summed the number of steps walked for each minute of walking to generate the minute-level cadence for each 1-minute segment of continuous walking during the intervention sessions. Because participants were allowed to take breaks during the intervention, we defined continuous walking as any 1-minute interval of sustained walking without interruption by standing or sitting. From the 1-minute segments of walking cadence, we calculated a 5-minute median across walking activities to generate the median walking cadence for each session (1–48) which was used for the analysis. We also calculated the number of breaks, defined as standing or sitting recorded by the accelerometer, and the total amount of time spent on breaks as measured in seconds for each session. The number of breaks and amount of time spent on breaks was used to compare the two groups during Phase 1. Walking segments that were less than 30 seconds were considered to be the start or end of a session or break and were excluded from the analysis. Cadence measured during the warm-up and cool-down periods as well as activities performed while standing in place (i.e., tapping and stepping) were excluded from analysis.

### Outcome

The primary outcome was improvement in 6MWT performance. A threshold of a change in 30-meters was chosen as this has been deemed a minimal clinically important distance (MCID) in multimorbid, frail older adults [36]. We chose this threshold conservatively and used the upper limit (30 meters) of the suggested range of MCID for the 6MWT. We calculated the change in 6MWT performance (4-month distance – baseline distance, meters) and used this as a dichotomous variable in our analysis.

### Statistical analysis

Data were accessed for research purposes from July 2023 to January 2024. Descriptive statistics including the mean and standard deviation (SD), median and interquartile range (IQR), and frequency were calculated for demographics, medical conditions, frailty, and objective measures of baseline physical function. We compared baseline characteristics using t-tests, Chi-square and Wilcoxon rank-sum tests where appropriate.

For our first objective we applied a group by time interaction to determine the impact of group on participant-level walking cadence during each of the three study phases. For this, we used a single stage model that included group interactions with each study phase indicator and included the random participant effects.


yij=(β1+v1i)P1j+(β2+v2i)P2j+(β3+v3i)P3j+(P1jxgroupi)β4+(P2jxgroupi)β5+(P3jxgroupi)β6+∈ij (1)

For Model 1 the time varying measurement of the walking cadence (y) of participant i (i = 1, 2, …, N) on occasion j (1, 2, …, ni) is predicted by P1j,P2j,P3j, which are indicator variables for the three study phases, respectively, which have fixed effects β1- β3, interaction effects β4- β6 and random participant effects v1i,v2i,v3i. The model also includes error term ∈ij.

For our second objective we first determined the overall association of group assignment on improvement in 6MWT MCID without the mediator of walking cadence. To do this we used a generalized linear model with a logistic link and controlled for baseline frailty status, age, highest level of education achieved, and sex.


logit(Y)=Groupβ1+Ageβ2+Educationβ3+Sexβ4+Frailβ4+βo (2)

To evaluate for the mediating effect of cadence we performed a two-stage model in which the random effects were estimated in the first stage to be used as predictors in a second-stage model. Specifically, the first stage model (i.e., Model 1 without the group by time interactions) examined the random participant effects of walking cadence across the three different phases (i.e., slope) of the intervention. From this model, the random effects were obtained and represent participant-level average cadence for each of the three phases. The second stage model included those random participant effects to determine if walking cadence across the three different phases were associated with improvement in functional capacity after the intervention as measured by 6-minute walk test MCID. For the model in Stage 1 we modelled time sequentially to mirror the three different phases of the intervention. We chose these three time periods as they identified distinct periods across the entirety of the intervention and would determine whether initial walking cadence (e.g., those participants with a slow cadence) may be more likely to improve their functional capacity.


yij=(β1+v1i)P1j+(β2+v2i)P2j+(β3+v3i)P3j+∈ij,(Stage1) (3)

For the Stage 1 model the time varying measurement of cadence (y) of participant i (i = 1, 2, …, N) on occasion j (1, 2, …, ni) is predicted by P1j,P2j,P3j, which are indicator variables for the three study phases, respectively, which have fixed effects β1- β3, and random participant effects v1i,v2i,v3i. The model also includes error term ∈ij.


logit(Yi)=∝+v0iB1+v1iB2+v2iB3+v3iB4+Frailβ5+Ageβ6+Educationβ7+Sexβ8(Stage2) (4)

In the Stage 2 single-level logistic regression model the participant-level outcome logit (Yi) is predicted by the constant, the random participant effects of Stage 1 for all 3 time periods across the study, and slope coefficients of other participant-level regressors that were apriori included due to their impact on response to exercise interventions. Initially we considered a 3-level model for the longitudinal analyses (Models 1 and 3) to account for the random center effects, in addition to the random participant effects. However, we compared the 2-level model (repeated observations within participants) to a 3-stage model (repeated observations within participants within centers) using the likelihood ratio test and there was no improvement in the 3-level model. Thus, for the longitudinal analyses, we presented results from the simpler 2-level model.

Finally, to generate an estimate for the number of steps a participant would need to increase from their baseline (Phase 1) to increase the odds of an improvement in functional capacity we performed a two-level model that mirrored Model 3 and Model 4. For this analysis we used variables to model the change in walking cadence from Phase 1 to Phase 2, and Phase 1 to Phase 3.


yij=(β1+v1i)P12j+(β2+v2i)P23+∈ij,(Stage1) (5)

Where P12 is the change in steps from phase 1 to phase 3 and P13 is the change from Phase 1 to Phase 3.


logit(Yi)=∝+v0iB1+v12iB2+v23iB3+Frailβ5+Ageβ6+Educationβ7+Sexβ8(Stage2) (6)

Using the variance from this model we estimated the change in cadence that would be necessary to increase the odds of improvement in functional capacity as assessed by the MCID in 6MWT distance after the intervention. All analyses were conducted using STATA v15.1 with a P value of < 0.05 used for statistical significance.

## Results

A total of 215 participants were screened for eligibility from the 14 enrolled retirement communities; 165 participants enrolled, and 102 participants had complete follow-up data and were included in the analysis. Table 1 shows the baseline characteristics among the participants included in the final analysis. There were no differences between the baseline characteristics of the HIW and CSW groups other than a higher percentage of female participants in the HIW group as compared to the CSW group. Importantly there were no differences in frailty classification, as measured by a categorical or continuous measure, or any of the other performance based functional measures.

**Table 1:** Patient Characteristics and Demographics.

Variables	CSW (n = 56)	HIW (n = 46)	P-value
Age	79.0 ± 8.0	78.7 ± 8.3	0.85
Gender:			0.02
Female	61 (71.8%)	71 (86.6%)	
Male	24 (28.2%)	11 (13.4%)	
Race:			0.69
White	66 (77.6%)	63 (76.8%)	
African American	17 (20.0%)	16 (19.5%)	
Non-White Hispanic	0 (0.0%)	2 (2.4%)	
Asian	1 (1.2%)	1 (1.2%)	
Other	1 (1.2%)	0 (0.0%)	
Highest Level of Education:			0.41
Grade school	1 (1.2%)	2 (2.4%)	
Some high school	4 (4.8%)	4 (4.9%)	
High school	10 (11.9%)	7 (8.5%)	
Some college	19 (22.6%)	14 (17.1%)	
College	19 (22.6%)	24 (29.3%)	
Masters or beyond	31 (36.9%)	31 (37.8%)	
Assistive Device:			0.27
None	30 (35.7%)	27 (32.9%)	
Cane	24 (28.6%)	18 (22.0%)	
Walker	23 (27.4%)	35 (42.7%)	
Scooter	1 (1.2%)	0 (0.0%)	
Wheelchair	0 (0.0%)	1 (1.2%)	
Other	6 (7.1%)	1 (1.2%)	
Mini-mental State Examination Total Score (SD)	28.3 ± 2.3	28.5 ± 2.4	0.68
SHARE-FI Frailty Score (SD):	2.4 ± 1.0	2.1 ± 1.1	0.120
SHARE-FI Frailty Category:			0.82
Frail	39 (45.9%)	41 (50.0%)	
Prefrail	46 (54.1%)	41 (50.0%)	
Cardiac Condition	32 (37.6%)	26 (31.7%)	0.75
High Blood Pressure	60 (71.4%)	65 (79.3%)	0.67
Diabetes	19 (22.6%)	18 (22.0%)	0.71
Asthma or other lung/ breathing problems	27 (32.1%)	24 (29.3%)	0.37
Epilepsy/ Seizures	1 (1.2%)	2 (2.4%)	0.44
Kidney Problems	7 (8.3%)	8 (9.8%)	0.74
Skin conditions	19 (22.6%)	16 (19.5%)	0.47
Blood disorder	6 (7.1%)	4 (4.9%)	0.82
Cancer	23 (27.4%)	23 (28.0%)	0.36
Syncope or blackouts	5 (6.0%)	6 (7.3%)	0.77
Stroke	11 (13.1%)	9 (11.0%)	0.39
Parkinson’s	2 (2.4%)	5 (6.1%)	0.05
Other conditions	33 (39.3%)	37 (45.1%)	0.65
Timed Up and Go Test (seconds) (SD)	18.5 ± 12.6	19.7 ± 14.0	0.66
Gait speed (maximal) meters/second (SD)	1.06 ± 0.27	1.05 ± 0.33	0.89
Gait speed (usual) meters/second (SD)	0.84 ± 0.22	0.81 ± 0.24	0.48
SPPB (SD)	6.7 ± 2.8	6.6 ± 2.8	0.85
6-minute walk test distance (meters) (SD)	255 ± 84	257 ± 94	0.93
Berg Balance Scale (SD)	41.5 ± 9.6	40.9 ± 11.2	0.79

**Fig 1:**
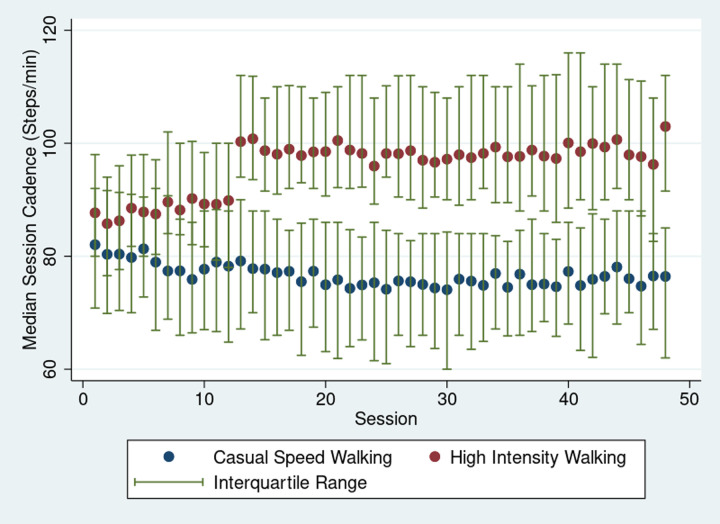
Median walking cadence and interquartile range across sessions for high-intensity and casual speed walking groups.

The median cadence across all three phases of the exercise sessions can be seen in Fig 1. The median (IQR) walking cadence for the CSW and HIW group was for Phase 1 (CSW 82[69, 91] vs. HIW 86[76, 94]), Phase 2 (CSW 77[64, 88] vs HIW 88[78, 97]), and Phase 3 (CSW 77[65, 86] vs HIW 100[88, 111]) (Table 2). The results for Model 1, evaluating difference in walking cadence across the three phases between the two groups, can be seen in S2 Table. There were no differences between the two groups during phase 1 where participants were instructed to walk at their own comfortable pace for 40 minutes. The CSW group took a higher number of breaks than the HIW group (mean(SD) CSW 25 ± 19 vs HIW 18 ± 16 (P = 0.003)); however, the total duration of those breaks in seconds was not different (mean(SD) CSW 1189 ± 1933 vs. HIW 1266 ± 3640 (P = 0.62)). Phase 2 demonstrated an increased participant-level walking cadence in the HIW as compared to the CSW group. To demonstrate fidelity of the intervention we found that participant-level walking cadence in Phase 3 for the HIW group was greater than that of the CSW group.

**Table 2:** Median walking cadence between groups during three intervention phases.

	Median Walking Cadence (Interquartile Range)		
Group (n = 102)	Phase 1	Phase 2	Phase 3
**Casual Speed Walking (n = 56)**	82(69, 91)	76(64, 88)	76(63, 85)
No change in 6MWT MCID (n = 34)	81(69, 92)	75(64, 85)	73(62, 82)
Improvement in 6MWT MCID (n = 22)	81(67, 90)	79(63, 90)	81(69, 88)
**High Intensity Walking** **(n = 46)**	87(77, 94)	89(79, 98)	100(90, 110)
No change in 6MWT MCID (n = 16)	83(77, 93)	86(78,96)	94(81,106)
Improvement in 6MWT MCID (n = 30)	88(76,96)	90(79,98)	103(94,114)

The baseline 6MWT among participants was not different between the two groups (Table 1). The mean (SD) distance walked during the 6MWT at the follow-up assessment was 265 ± 97 meters in the CSW group and 315 ± 100 for the HIW group. The number of participants that reached the primary outcome, change in MCID from baseline 6MWT, was 39% (22/56) for the CSW group as compared to 65% (30/46) for HIW group. In the baseline model (Model 2), participants in the HIW group demonstrated more than a 3-fold increase in the odds of 6MWT improvement (OR 3.23, 95% CI 1.34, 7.78; P-0.009) as compared to the CSW when controlling for baseline frailty, sex, education, and age (S3 Table).

The results of Model 3, to evaluate the participant-level effects of walking cadence on change in MCID can be seen in S4 Table (Stage 1) and the results of Model 4 can be seen in Table 3. The participant-level random effects in the stage 2 model for Phase 1 and Phase 2 were not associated with an increase in the odds of 6MWT improvement. An increase in participant-level cadence during phase 3 was associated with an increased odds of improvement in 6MWT (OR: 0.11, 95% CI: 0.033, 0.18; p = 0.005). The group effect in this model was no longer significant when accounting for participant-level changes in walking cadence across the three study phases. This result suggests that the group effect was mediated by an increase in participant-level walking cadence, specifically during Phase 3 of the intervention. The results of Model 5 and Model 6 (S5 Table) demonstrate a similar effect size (OR: 0.10, 95% CI: 0.035, 0.17; p = 0.003) to Model 4. Using the variance estimate for the change in walking cadence from Phase 1 to Phase 3 from Model 5, participants that were able to increase their walking cadence by 14 steps/min had a 10% increase in the odds of improvement of their 6MWT by the MCID.

**Table 3:** Second stage of mixed effects logistic regression to determine walking cadence on improvement in 6MWT distance.

Variable	Coef.	Std. Err.	P>|z|	[95% Conf. Interval]	
Phase 1	-0.012	0.0762684	0.878	-0.16	0.14
Phase 2	-0.070	0.0812506	0.390	-0.23	0.09
Phase 3	0.11	0.0375667	0.005	0.033	0.18
Treatment Group (Ref: CSW)	-0.20	0.6980271	0.773	-1.57	1.17
SHARE-FI Frailty category (Ref: prefrail)	0.59	0.4968611	0.236	-0.39	1.56
Age	-0.033	0.035319	0.357	-0.10	0.037
Sex (Ref: female)	0.56	0.6179731	0.366	-0.65	1.77
Education (Ref: Some high-school)	0.26	0.213864	0.233	-0.16	0.67
_cons	0.90	2.918003	0.758	-4.82	6.62

## Discussion

Our results demonstrate that increased participant-level walking cadence was associated with a 10 percent increase in the odds of functional capacity improvement in prefrail and frail older adults. Prefrail and frail older adults were able to increase and sustain their walking cadence for a duration of 30 minutes and in doing so improved their functional outcome. To increase the intensity of walking, participants in the HIW group were instructed and encouraged to walk “as fast as you safely can,” beginning in Phase 2 and throughout Phase 3. Thus, we expected to see changes in walking cadence in the HIW group to increase walking speed during these phases of the intervention. The overall exercise dose (frequency, duration, and intensity) between the two groups only differed with respect to the intensity component as frequency and duration were kept constant between the two groups [14]. The increased odds of improvement in functional capacity in the HIW group was mediated by the increased activity intensity as measured through participant-level walking cadence. Thus, prefrail and frail older adults engaged in walking interventions can derive further improvement in their functional outcomes by increasing cadence during a fixed volume of walking exercise.

Our finding that walking cadence can serve as a surrogate measure of activity intensity is consistent with prior literature. Tudor Locke et al recently demonstrated in the CADENCE-Adults study that a walking cadence > 100 steps/min is associated with moderate intensity activity, equivalent to ≥ 3 metabolic equivalents [29]. The mean walking cadence for the HIW group throughout our study was 100 steps/min, and this group experienced a greater proportion of participants who reached a 6MWT MCID. However, what may be more important than reaching a specific cadence threshold (i.e., 100 steps/min) may be the increase in walking cadence between comfortable walking pace and the “walk as fast as you safely can,” pace as implemented during Phase 3. Our analysis was not focused on evaluating a specific cadence target but rather whether an increase in participant-level cadence from their baseline comfortable walking pace during Phase 1 improved functional capacity. We demonstrated that an increase of 14 steps/min during the intervention sessions increased the odds of an improvement in functional capacity. Future studies will be needed to evaluate whether interventions that target an absolute cadence threshold (i.e., ≈ 100 steps/min) or relative change in cadence from usual pace has a greater impact on the odds of improvement in functional outcomes.

Our study has important strengths. Notably we used objectively measured walking cadence during the intervention itself from a thigh worn activPAL accelerometer. Thigh worn accelerometers, such as the activPAL, have demonstrated increased accuracy for step counts as compared to wrist worn devices, and are even accurate at low speeds (<0.5 m/s) commonly encountered in prefrail and frail older adults [37–39]. Our study provides a comprehensive measurement of walking cadence during a walking intervention and contributes to the growing evidence supporting its use as a measure of activity intensity in walking interventions. For our analysis we chose clinically relevant outcomes and analyzed participant-level data. Our analysis used an objectively measured primary outcome of MCID in 6MWT distance that is a reliable, well validated, and clinically important outcome to prefrail and frail older adults [36]. Our approach to the analysis focused on participant-level changes in walking cadence as compared to a group or threshold (i.e., 100 steps/min) level change. These results of our analysis suggest participant-level changes in walking cadence from their comfortable walking pace were the important mediator to the increased odds of functional improvement. This finding has significant clinical implications as cadence can be measured during walking interventions and used as a guide to enhance current exercise volumes at the patient level. Thus, participant-level cadence targets during supervised exercise can be used to increase the odds of functional improvements in prefrail and frail older adults.

Despite the strengths of our study, we note several limitations. Participants were actively recruited from retirement communities and so were motivated to participate in an activity intervention and may not be fully representative of all prefrail and frail older adults in retirement communities. Nonetheless, baseline 6MWT distance for the HIW and CSW groups was roughly 250 meters which is well below community based norms for older adults and highlights the significantly decreased functional capacity of the study cohort [40]. Participants in the CSW group completed the protocol at a higher frequency than those participants in the HIW group and there may have been a selection bias in the HIW group. However, we noted that there were no differences identified in walking cadence during Phase 1 where participants selected their comfortable walking speed or any of the initial baseline functional assessments. Our intervention was supervised and implemented by research assistants who provided scripted motivational prompts to participants in both treatments. It is unclear whether participants would achieve the same increase in walking cadence and improvement in functional capacity without the supervision and encouragement provided by the research assistants. Thus, we cannot infer whether prefrail and frail older adults that increase walking cadence during a walking program without supervision would obtain similar benefits.

## Conclusions

In conclusion, we demonstrated that in prefrail and frail older adults walking cadence can serve as an objective, clinically relevant measure of walking intensity. Participants were able to significantly increase their cadence during a 12-week walking intervention, which was associated with higher odds of improving 6MWT distance beyond the MCID. Participant-level increases in walking cadence above their comfortable walking pace, or approximately an increase in 14 steps/minute, produced clinically important improvements in functional capacity among prefrail and frail older adults. Future studies should explore optimal cadence protocols and achievable targets for older adults, as well as the effectiveness of real-time feedback in helping participants reach and maintain those targets.

## Supporting Information

S1 Table Summary Protocol for High-Intensity and Casual Speed Walking Groups. (DOCX)

S2 Table Comparison of walking cadence across phases of the intervention (Model #1). (DOCX)

S3 Table Baseline model logistic regression (Model #2). (DOCX)

S4 Table Participant-level random effects of walking cadence across each intervention phase. (DOCX)

S5 Table Change in walking cadence between phases. (DOCX)
