# Supplementary material for: Walking cadence as a measure of activity intensity and impact on functional capacity for prefrail and frail older adults
Source: PLoS One. 2025 Jul 16;20(7):e0323759. doi: 10.1371/journal.pone.0323759 (PMC12266393; doi:10.1371/journal.pone.0323759)
Supplement: S1 Table — (DOCX) [file pone.0323759.s001.docx]

**Table 1:** Summary Protocol for High-Intensity and Casual Speed Walking Groups

| Phase | HIW group | CSW group |
| --- | --- | --- |
| Phase 1  (Week 1) | Week 1: Walking at a casual, self-selected pace for 45 minutes (rest breaks as needed) | Walking at a casual, self-selected pace for 45 minutes (rest breaks as needed) |
| Phase 2  (Week 2-4) | Week 2: Walking at ~50% HRmax for 40 minutes + 5 minutes of stairs (2.5 minutes at beginning and 2.5 minutes at end)  Week 3: Walking at ~60% HRmax for 40 minutes + 5 minutes of stairs (2.5 minutes at beginning and 2.5 minutes at end)  Week 4: Walking at ~70% HRmax for 40 minutes + 5 minutes of stairs (2.5 minutes at beginning and 2.5 minutes at end) | Walking at casual, self-selected pace for 40 minutes + 5 minutes of stairs (2.5 minutes at beginning at 2.5 minutes at end) |
| Phase 3  (Week 5-12) | **Activity/Intensity:**   - 10-minute warm up on level surfaces with gradual increase in intensity - 2.5 min stepping “as fast as you safely can” - 5-minute walking tasks “as fast as you safely can” - 2.5 min stepping “as fast as you safely can” - 5-minute walking tasks “as fast as you safely can” - 5-min walking with ankle weight “as fast as you safely can” - 5-min walking variable directions “as fast as you safely can” - 5-min walking with ankle weight “as fast as you safely can” - 5-min cool down “as fast as you safely can” | **Activity/Intensity**   - 10-minute warm up on level surfaces at comfortable pace throughout - 2.5 min stepping “relaxed, comfortable pace” - 5-minute walking tasks “relaxed, comfortable pace” - 2.5 min stepping “relaxed, comfortable pace” - 5-minute walking tasks “relaxed, comfortable pace” - 5-min walking with ankle weight “relaxed, comfortable pace” - 5-min walking variable directions “relaxed, comfortable pace” - 5-min walking with ankle weight “relaxed, comfortable pace” - 5-min cool down |
