# Supplementary material for: Walking cadence as a measure of activity intensity and impact on functional capacity for prefrail and frail older adults
Source: PLoS One. 2025 Jul 16;20(7):e0323759. doi: 10.1371/journal.pone.0323759 (PMC12266393; doi:10.1371/journal.pone.0323759)
Supplement: S2 Table — (DOCX) [file pone.0323759.s002.docx]

**Table 2: Comparison of walking cadence across phases of the intervention (Model #1)**

| Variable | Coef. | Std. Err. | P>\|z\| | [95% Conf. Interval] | |
| --- | --- | --- | --- | --- | --- |
| Phase 1 cadence (mean) | 78.81 | 2.47 | <0.001 | 73.96 | 83.65 |
| Phase 2 cadence (mean) | 75.54 | 2.59 | <0.001 | 70.47 | 80.62 |
| Phase 3 cadence (mean) | 75.10 | 2.56 | <0.001 | 70.08 | 80.12 |
| Treatment Group x Phase 1 cadence interaction | 5.60 | 3.52 | 0.11 | -1.31 | 12.51 |
| Treatment Group x Phase 2 Cadence interaction | 11.14 | 3.71 | <0.001 | 3.88 | 18.41 |
| Treatment Group x Phase 3 Cadence interaction | 22.39 | 3.66 | <0.001 | 15.23 | 29.56 |

Model 1 is a mixed effects linear regression with a group by time interaction to determine the impact of group on participant-level walking cadence during each of the three study phases. For this, we used a single stage model that included group interactions with each study phase indicator and included the random subject effects.

$yij=\left( \beta1+ v1i \right)P1j+\left( \beta2+ v2i \right)P2j+\left( \beta3+ v3i \right)P3j+\left( P1j x groupi \right)\beta4+\left( P2j x groupi \right)\beta5+\left( P3j x groupi \right)\beta6+ \epsilon ij$ (Model 1)

Phase 1-Phase 3 represents the mean cadence if the CSW group across the three different phases of the intervention. The Group Phase 1-Group Phase 3 variables represent the difference in cadence between the CSW and HIW groups. There was no difference between groups during phase 1. The HIW increased their cadence in phase 2 and phase 3 as compared to the CSW group.
