# Supplementary material for: Walking cadence as a measure of activity intensity and impact on functional capacity for prefrail and frail older adults
Source: PLoS One. 2025 Jul 16;20(7):e0323759. doi: 10.1371/journal.pone.0323759 (PMC12266393; doi:10.1371/journal.pone.0323759)
Supplement: S3 Table — (DOCX) [file pone.0323759.s003.docx]

**Table 3: Baseline model logistic regression (Model #2)**

| Independent Variable | Odds Ratio | Std. Err. | P-value | [95% Conf. Interval] | |
| --- | --- | --- | --- | --- | --- |
| Group  (Ref: CSW) | 3.11 | 1.37 | 0.01 | 1.32 | 7.37 |
| Frail  (Ref: Prefrail) | 1.54 | 0.67 | 0.32 | 0.66 | 3.60 |
| Age  (Continuous) | 0.99 | 0.03 | 0.75 | 0.94 | 1.05 |
| Sex  (Ref: Female) | 1.71 | 0.90 | 0.31 | 0.60 | 4.81 |
| Education level  (Ref: Some high school) | 1.14 | 0.21 | 0.47 | 0.80 | 1.63 |
| _cons | 0.44 | 0.97 | 0.71 | 0.01 | 33.03 |

Model 2 is a logistic regression on change in 6MWT distance (>30 meters MCID). The independent variables include group (reference CSW), frailty category (reference prefrail), age (continuous), sex (reference female) and education level (reference some high school). Participants in the HIW group had an increase in the odds of an improvement in 6MWT distance as compared to the CSW group.
