# Supplementary material for: Walking cadence as a measure of activity intensity and impact on functional capacity for prefrail and frail older adults
Source: PLoS One. 2025 Jul 16;20(7):e0323759. doi: 10.1371/journal.pone.0323759 (PMC12266393; doi:10.1371/journal.pone.0323759)
Supplement: S4 Table — (DOCX) [file pone.0323759.s004.docx]

**Table 4: Participant-level random effects of walking cadence across each intervention phase.**

| Variable | Coef. | Std. Err. | | z | | P>\|z\| | [95% Conf. | Interval] |
| --- | --- | --- | --- | --- | --- | --- | --- | --- |
| Phase 1 | 81.29 | 1.48 | | 55.08 | | 0.00 | 78.40 | 84.18 |
| Phase 2 | 80.51 | 1.65 | | 48.70 | | 0.00 | 77.27 | 83.75 |
| Phase 3 | 85.25 | 1.90 | | 44.84 | | 0.00 | 81.52 | 88.98 |
| Random-effects Parameters | | | Estimate | | Std. Err. | | [95% Conf. | Interval] |
| individual: Unstructured var(p1)  var(p2)  var(p3)  cov(p1,p2)  cov(p1,p3)  cov(p2,p3) | | | 205.202 273.1013 365.2609 222.3513 191.214 255.9185 | | 31.18523 39.04017 51.33554 33.06154 34.06882 40.53615 | | 152.3427 206.3685 277.3136 157.5519 124.4403 176.4691 | 276.4022 361.4133 481.0998 287.1507 257.9876 335.3679 |
| var(Residual) | | | 48.48261 | | 0.8029992 | | 46.93403 | 50.08229 |

Model 3 is the first stage model to determine if changes in participant level walking cadence are associated with improvement in functional capacity.

$yij=\left( \beta1+ v1i \right)P1j+\left( \beta2+ v2i \right)P2j+\left( \beta3+ v3i \right)P3j+ \epsilon ij,$ (Stage 1) (Model 3)
