# Supplementary material for: Walking cadence as a measure of activity intensity and impact on functional capacity for prefrail and frail older adults
Source: PLoS One. 2025 Jul 16;20(7):e0323759. doi: 10.1371/journal.pone.0323759 (PMC12266393; doi:10.1371/journal.pone.0323759)
Supplement: S5 Table — (DOCX) [file pone.0323759.s005.docx]

**Table 5: Change in walking cadence between phases**

**First stage:**

| Variable | Coef. | Std. Err. | | z | | P>\|z\| | [95% Conf. Interval] | |
| --- | --- | --- | --- | --- | --- | --- | --- | --- |
| Phase 1 to Phase 2 | -0.776487 | 0.742394 | | -1.05 | | 0.296 | -2.231554 | 0.6785796 |
| Phase 1 to Phase 3 | 3.96125 | 1.42950 | | 2.77 | | 0.006 | 1.159473 | 6.763035 |
| _cons | 81.2879 | 1.47587 | | 55.08 | | 0.000 | 78.39527 | 84.18058 |
| Random-effects Parameters | | | Estimate | | Std. Err. | | [95% Conf. | Interval] |
| individual: Unstructured var(p12) var(p13)  var(_cons)  cov(p12,p13)  cov(p12,_cons)  cov(p13,_cons) | | | 33.60028 188.0333 205.2007 47.55454 17.14891 -13.98825 | | 7.835658 29.13833 31.18725 12.4856 11.12344 21.51698 | | 21.2736 138.7809 152.3385 23.08321 -4.652632 -56.16077 | 53.0695 254.7652 276.4063 72.02587 38.95045 28.18426 |
| var(Residual) | | | 48.48263 | | 0.8029997 | | 46.93405 | 50.0823 |

**Second stage:**

| Variable | Coef. | Std. Err. | P>\|z\| | [95% Conf. Interval] | |
| --- | --- | --- | --- | --- | --- |
| Phase 1-2  **Phase 1-3**  intercept Frail Category Age  Sex  Education _cons | -.0707711 .1022402 .0236992 .5766182 -.0311948 .5912792 .2398264 .7856462 | .0810764 .034294 .0199309 .4949339 .0348883 .6046125 .2069079 2.869574 | 0.383 0.003 0.234 0.244 0.371 0.328 0.246 0.784 | -.2296778 .0350251 -.0153647 -.3934344 -.0995747 -.5937394 -.1657056 -4.838615 | .0881356 .1694552 .0627631 1.546671 .0371851 1.776298 .6453584 6.409908 |
| **Center** var(_cons) | 0.2332823 | 0.3702301 |  | 0.0103988 | 5.23334 |

Model 5 and Model 6 evaluate the change participant-level change in walking cadence from Phase 1 to Phase 2 and Phase 1 to Phase 3. For those participants who increased their cadence in phase 3 there was a roughly 10% increase in the odds of improvement of functional capacity. Using the variance estimate from Model 5 (var(p13)=188)), an increase of 14 steps/min from comfortable walking pace resulted in a 10% odds increase.
